# Impact of Oseltamivir and Diabetes Development

**DOI:** 10.3390/ph18010128

**Published:** 2025-01-18

**Authors:** Bor-Show Tzang, Chih-Chen Tzang, Pei-Hua Chuang, I-Ying Kuo, Yu-Chun Pan, Pei-Hsun Wu, Tsai-Ching Hsu

**Affiliations:** 1 Department of Biochemistry, School of Medicine, Chung Shan Medical University, Taichung 402, Taiwan; bstzang@csmu.edu.tw; 2 Institute of Medicine, Chung Shan Medical University, Taichung 402, Taiwan; dylan.ymu@gmail.com; 3 Department of Clinical Laboratory, Chung Shan Medical University Hospital, Taichung 402, Taiwan; 4 Immunology Research Center, Chung Shan Medical University, Taichung 402, Taiwan; 5 School of Medicine, College of Medicine, National Taiwan University, Taipei City 100, Taiwan; jerrytzang@gmail.com (C.-C.T.); b09401054@ntu.edu.tw (Y.-C.P.); b10401070@g.ntu.edu.tw (P.-H.W.); 6 Department of Biotechnology, College of Biomedical Science, Kaohsiung Medical University, Kaohsiung 807, Taiwan; iyingkuo@kmu.edu.tw

**Keywords:** antiviral therapy, neuraminidase inhibitors, retrospective cohort study, Taiwan National Health Insurance Research Database

## Abstract

**Background/Objectives**: Influenza is a major global health challenge, causing thousands of deaths annually. Antiviral drugs, particularly oseltamivir, a neuraminidase inhibitor, have become essential therapeutic options due to their oral bioavailability and efficacy. Previous studies suggest a potential association between oseltamivir use and the onset of diabetes mellitus. However, further investigation is needed to establish a definitive link. **Methods**: This retrospective cohort study utilized data from the Taiwan National Health Insurance Research Database (NHIRD), including 1,631,968 patients (815,984 oseltamivir users) between 1 January 2009 and 28 December 2018. All statistical analyses were performed using SAS 9.4M8 software (SAS Institute Inc., Cary, NC, USA). **Results**: Cox proportional hazards regression and multivariate analyses revealed a statistically significant association between oseltamivir use and overall diabetes risk (HR = 1.027, *p* = 0.0186). While no significant association was observed for Type 1 diabetes (HR = 1.021; *p* = 0.06795), oseltamivir users showed a higher incidence of Type 2 diabetes (HR = 1.024; *p* < 0.05). Oseltamivir was also linked to increased risks of comorbidities, including dyslipidemia (HR = 1.295, *p* < 0.0001), chronic liver disease (HR = 1.446, *p* < 0.0001), hypertension (HR = 1.586, *p* < 0.0001), and obesity (HR = 2.949, *p* < 0.0001). **Conclusions**: Oseltamivir is associated with an increased risk of Type 2 diabetes but not Type 1, and related comorbidities.

## 1. Introduction

Influenza poses a significant global health threat, accounting for 3 to 5 million severe cases and 290,000 to 650,000 deaths annually, making it an important contributor to worldwide morbidity and mortality [1]. Vaccination remains the cornerstone of influenza prevention; however, its efficacy is often constrained, particularly during periods of anti-genic drift [2,3]. This limitation can result in significant outbreaks, particularly among vulnerable populations [4], as the timely availability of strain-specific vaccines is frequently challenging [5,6]. This underscores the pivotal role of antiviral drugs in influenza management. Various antiviral agents have been developed to inhibit viral replication by targeting key components of the influenza virus, including neuraminidase, hemagglutinin, M2 ion channels, nucleoproteins, and RNA-dependent RNA polymerases [7,8]. Among them, neuraminidase inhibitors (NAIs) have emerged as a notable innovation since their development in the 1990s for prophylaxis and treatment [9]. As neuraminidase is a crucial enzyme responsible for releasing newly formed virions from host cells, its inhibition effectively hinders the spread of the virus within the respiratory tract [10,11].

Oseltamivir, namely Tamiflu^®^ (Welwyn Garden City, UK), a widely recognized NAI, is notable for its oral bioavailability, efficacy, and well-established safety profile [5]. As an oral prodrug, it is metabolized into its active form, oseltamivir carboxylate (OC), which inhibits the neuraminidase enzyme of influenza viruses. By blocking this enzyme, OC prevents the release of new viral particles from infected cells, reducing viral replication and mitigating the severity and duration of influenza symptoms when administered within 48 h of onset [12]. Prophylactic use can also prevent symptomatic illness and complications such as bronchitis and pneumonia. Early administration has been shown to shorten symptom duration, reduce viral shedding, and lower the risk of influenza-related complications [13,14,15]. Oseltamivir’s straightforward pharmacokinetics and low potential for drug–drug interactions allow safe oral administration at doses up to 1000 mg daily in healthy individuals [16,17]. Compared to placebo, oseltamivir has been associated with an increased risk of nausea, vomiting, and composite gastrointestinal symptoms, though the risk of diarrhea appears lower [18]. However, adverse effects such as gastrointestinal discomfort and rare reports of renal and psychiatric syndromes underscore the need for further investigation into its broader safety profile [19]. Diabetes Mellitus (DM) is characterized by hyperglycemia, impaired immune function, and an elevated risk of infection [20]. Despite similar clinical manifestations, DM is classified into two primary types: Type 1 diabetes, marked by insufficient insulin production, and Type 2 diabetes, characterized by insulin resistance [21]. Managing influenza in patients with DM requires careful consideration, as they are at an increased risk of severe illness and poorer prognoses [22]. Consequently, NAIs are prescribed more frequently [23] and promptly [24] to this population in clinical practice. Retrospective studies further support the use of oseltamivir in diabetic patients with influenza, demonstrating improved outcomes, including a reduced risk of respiratory complications and lower hospitalization rates [25].

There is limited research exploring the association between oseltamivir and DM development. Animal studies suggest that higher doses of oral oseltamivir may increase glucose levels in rats [26]. Reported adverse event profiles among clinical trials and case reports have documented instances of hyperglycemia in influenza patients treated with oseltamivir [27,28]. At the molecular level, evidence indicates that oseltamivir may interfere with the insulin signaling pathway [29], potentially disrupting glucose metabolism. This disruption could lead to hyperglycemia, thereby increasing the risk of developing diabetes. However, the clinical information for oseltamivir use and human DM remains lacking. Therefore, our retrospective study reviewed and analyzed data from a large-scale nationwide cohort of the Taiwan National Health Insurance Research Database (NHIRD) to explore the association between oseltamivir and the risk of diabetes.

## 2. Results

### 2.1. Participant’s Characteristics

**Figure 1:**
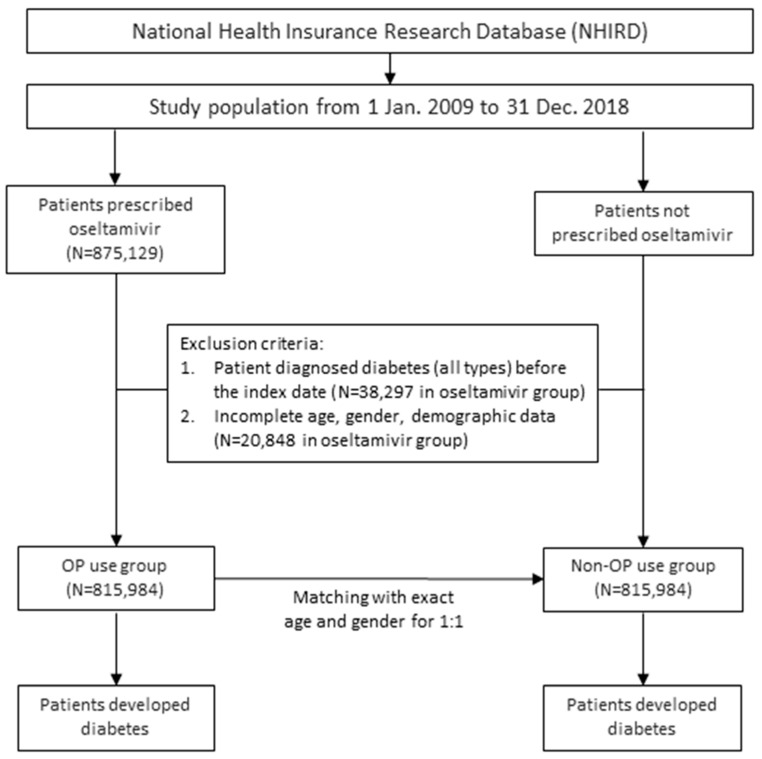
Flowchart of participants.

Figure 1 presents a flowchart of the study participants. From the registry, a total of 875,129 patients were identified as oseltamivir users. Among these, 38,297 patients were excluded due to a prior diagnosis of diabetes, and an additional 20,848 were excluded due to incomplete demographic data, including age and gender. As a result, 815,984 patients remained in the oseltamivir group for the final analysis. A control group was generated through age- and gender-matching with the oseltamivir group in a 1:1 ratio, resulting in 1,631,968 patients in the subsequent analysis.

Table 1 displays the baseline characteristics and the prevalence of comorbidities and outcomes for both oseltamivir and control groups, with comparisons made using chi-squared tests. The oseltamivir group had significantly higher rates of dyslipidemia, chronic liver disease, hypertension, coronary artery disease (CAD), cerebrovascular disease, obesity, Chronic Kidney Disease (CKD), malignancy, human immunodeficiency virus infection (HIV), and autoimmune diseases, all with *p* < 0.001.

Additionally, the crude occurrence of all types of diabetes (2.62% vs. 2.36%, *p* < 0.0001) and Type 2 diabetes (2.53% vs. 2.29%, *p* < 0.0001) was significantly higher in the oseltamivir group compared to the control group. However, there was no significant difference in the crude occurrence of Type 1 diabetes (0.11% vs. 0.10%, *p* = 0.1293). The mean follow-up period for both groups was also calculated, with oseltamivir patients showing a slightly longer follow-up period for all types of diabetes (6.8984 vs. 6.9132 years, *p* = 0.0035) and Type 2 diabetes (6.9023 vs. 6.9166 years, *p* = 0.0045). There was no significant difference in the follow-up period for Type 1 diabetes between groups (6.9979 vs. 6.9983 years, *p* = 0.9247).

### 2.2. Relative Hazard Ratios for Diabetes in Oseltamivir and Non-Oseltamivir Groups

With an HR of 1.027 and a *p*-value of 0.0186, the analysis indicates a statistically significant increase in all types of diabetes in the oseltamivir group compared to the control group after adjusting for all comorbidities (Table 2). After adjusting for oseltamivir use and various comorbidities, several conditions were associated with an increased risk of DM. Specifically, patients with dyslipidemia (HR = 1.295, *p* < 0.0001), chronic liver disease (HR = 1.446, *p* < 0.0001), hypertension (HR = 1.586, *p* < 0.0001), and obesity (HR = 2.949, *p* < 0.0001) demonstrated a higher likelihood of developing diabetes compared to those without these conditions.

Table 3 shows no significant difference in the HR for Type 1 diabetes after adjusting for all comorbidities (HR = 1.021, *p* = 0.6795). Following adjustment for oseltamivir use and comorbidities, hypertension (HR = 1.78, *p* < 0.0331) remained a significant contributor to DM risk.

Table 4 shows a slight increase in the HR for Type 2 diabetes after adjusting for all comorbidities (HR = 1.024, *p* = 0.037). Following adjustment for oseltamivir use and comorbidities, dyslipidemia (HR = 1.306, *p* < 0.0001), chronic liver disease (HR = 1.463, *p* < 0.0001), hypertension (HR = 1.607, *p* < 0.0001), and obesity (HR = 2.8, *p* < 0.0001) remained significant contributors to DM risk. Conversely, cerebrovascular disease was associated with a reduced risk of Type 2 diabetes (HR = 0.84, *p* = 0.0296).

## 3. Discussion

Using a nationwide population-based health database, we compared patients with and without prior use of oseltamivir. After matching for age and gender and adjusting for comorbidities, our analysis revealed an association between the use of this anti-influenza medication and a higher incidence of Type 2 diabetes, but no association was observed with Type 1 diabetes. This finding, derived from a large-scale retrospective national cohort, underscores a potential link that warrants further investigation to elucidate the underlying mechanisms.

Oseltamivir may possess diabetogenic effects by impacting insulin signaling pathways and metabolic processes, potentially explaining its association with a higher incidence of Type 2 diabetes. Research has demonstrated that oseltamivir inhibits neuraminidase-1 (Neu-1), an enzyme that forms part of the insulin receptor–signaling platform in conjunction with matrix metalloproteinase-9 (MMP-9). Neu-1 is essential for activating the insulin receptor, and its inhibition leads to impaired insulin signaling and reduced glucose metabolism [29]. This disruption in insulin receptor activation can have significant metabolic consequences, particularly for individuals at risk of or already suffering from metabolic disorders such as Type 2 diabetes. In support of this, animal studies suggest that oseltamivir may have diabetogenic effects, including the onset of hyperglycemia, worsening of pre-existing diabetes, and even the potential induction of new diabetes cases [26].

Oseltamivir has been associated with diabetogenic effects, potentially due to its inhibition of neuraminidase-1 (Neu-1), an enzyme critical for activating insulin receptors in conjunction with matrix metalloproteinase-9 (MMP-9). [26,29]. Neu-1 plays a central role in regulating glucose metabolism, insulin sensitivity, adipocyte hypertrophy, and hepatic lipid metabolism. Its inhibition disrupts insulin receptor signaling, leading to impaired glucose uptake and hyperglycemia, particularly in individuals predisposed to Type 2 diabetes or metabolic disorders [29,30]. Animal studies highlight Neu-1’s role in obesity, insulin resistance (IRES), and non-alcoholic fatty liver disease (NAFLD) by regulating adipocyte hypertrophy, insulin receptor (IR) signaling, and hepatic lipid metabolism [26,30,31,32]. Studies using sialidase inhibitors like 2-deoxy-2,3-dehydro-N-acetylneuraminic acid (DANA) and animal models with Neu-1 knockout or overexpression show that inhibiting Neu-1 leads to hyperglycemia and impaired glucose tolerance [31,33]. Clinically, observational studies associate oseltamivir with hyperglycemia, worsening glycemic control [26], and delayed-onset adverse events, including neuropsychiatric symptoms and metabolic disturbances, suggesting that its inhibition of Neu-1 disrupts metabolic homeostasis [26,34].

Furthermore, a case report highlighted transient hyperglycemia in children following oseltamivir administration, suggesting that the drug’s effects on glucose metabolism may not be limited to adults or those with pre-existing conditions [27]. However, the short-term nature of its administration raises questions about the extent to which it may contribute to the observed metabolic disturbances. Further research is needed to assess whether oseltamivir has lasting effects on glucose metabolism, especially in populations already at risk of diabetes.

However, the observed association between Type 2 diabetes risk and oseltamivir use may reflect confounding factors linked to differing care strategies for high-risk populations. Type 2 diabetes is a multifactorial metabolic disorder influenced by various risk factors, including advancing age, African American or Asian ethnicity, male sex, obesity, and insulin resistance [35,36]. Managing influenza in diabetic patients requires particular care, as they are 4.72 times more likely to require intensive care compared to non-diabetic individuals [22]. Early administration of NAIs, including oseltamivir, is a standard intervention in such cases, as it has been shown to reduce hospitalization duration and is often recommended for diabetic patients [24]. Moreover, certain high-risk diabetes subgroups, such as obese patients, receive intense medical attention due to their increased susceptibility to severe influenza complications. These patients are frequently prescribed antivirals earlier than non-obese individuals [37], potentially contributing to the observed association. Another confounding factor is that diabetic patients are more susceptible to infections, including influenza [38]. Many of these individuals may have undiagnosed health conditions such as chronic inflammation, obesity, or prediabetes that independently increase their risk for Type 2 diabetes, complicating efforts to establish a direct causal link between oseltamivir use and diabetes onset [35]. Additionally, it is important to consider whether influenza infection, rather than antiviral treatment, contributes to the observed higher incidence of Type 2 diabetes among previously infected individuals. The physiological impacts of influenza, including glycemic variability and chronic inflammation, may not only exacerbate disease severity in individuals with existing diabetes but could also increase the risk of Type 2 diabetes development in those predisposed to the condition [39].

Although our investigation links oseltamivir to a higher incidence of Type 2 diabetes, no evidence suggests that it influences the development of Type 1 diabetes. Mechanistically, Type 1 diabetes arises from the autoimmune destruction of pancreatic beta cells, distinct from the insulin resistance that characterizes Type 2 diabetes [40,41]. The pathophysiology and risk factors of Type 1 diabetes, including genetic predisposition, viral infections, and environmental triggers, suggest that antiviral drugs like oseltamivir are unlikely to influence its development [42,43]. Viral infections, however, may contribute to Type 1 diabetes through direct islet cell infection, immune modulation, and increased beta cell stress [44]. For example, a nationwide cohort study in Norway, analyzing data from over 2.5 million residents under the age of 30, reported a twofold increase in the risk of Type 1 diabetes following laboratory-confirmed H1N1 infection. Interestingly, the study found no significant increase in risk among individuals with only clinically diagnosed infections [45]. Additionally, influenza viruses have been shown to infect pancreatic cells both in vitro and in vivo, potentially triggering inflammatory responses or impairing insulin secretion [46]. Type 1 diabetes is characterized by elevated levels of pro-inflammatory cytokines, such as IL-6, TNF-α, and IL-1β, typically transient and linked to the acute immune response against the virus [47]. In contrast, type 2 diabetes is associated with obesity and metabolic syndrome, where sub-acute chronic inflammation is driven by pathways like stress-activated Jun N-terminal kinases (JNK), and NF-κB plays a key role [48,49]. This inflammation is further amplified by adipokines, exacerbating metabolic dysfunction [50]. Oseltamivir, however, appears to exert its effects directly through intrinsic Neu receptor modulation without prominent activation of cytokine pathways.

There is also a documented case of a Type 2 diabetes patient who developed Type 1 diabetes following influenza infection and oseltamivir therapy [51]. The patient’s Type 2 diabetes had been well-controlled. Two months after receiving a diagnosis of influenza and treatment with oseltamivir, the patient exhibited significantly elevated fasting plasma glucose and glycated hemoglobin (HbA1c) levels. Further investigation revealed the presence of anti-glutamic acid decarboxylase (anti-GAD) antibodies, indicative of autoimmune diabetes, and HLA typing identified alleles commonly associated with Type 1 diabetes susceptibility. This case highlights the complex interplay between influenza, antiviral therapy, and diabetes pathogenesis, warranting further research into these mechanisms.

While the effects of oseltamivir on diabetes require further exploration, other less-researched associations warrant attention. Emerging evidence suggests that oseltamivir use may reduce the risk of certain cancers, including liver cancer, and is associated with lower cancer-related mortality in population-based studies [52]. In vitro studies have demonstrated that oseltamivir decreases the viability, migration, and invasion of Huh-7 and HepG2 liver cancer cells while in vivo, it significantly inhibited tumor growth in a xenograft mouse model, indicating its potential as an alternative therapeutic strategy [53]. Furthermore, oseltamivir has been linked to a reduced risk of stroke or transient ischemic attack following influenza, particularly among younger individuals and older adults [54]. Another population-based study revealed that oseltamivir treatment significantly lowers the risk of recurrent cardiovascular events in patients with pre-existing cardiovascular disease, demonstrating a substantial protective effect [55]. This effect is likely due to reduced proinflammatory cytokines, inflammation, viral load, and illness duration, mitigating influenza-associated thrombotic risks [6]. These findings suggest that further research is needed to fully understand oseltamivir’s broader effects.

Several limitations of this study must be acknowledged. First, the retrospective design of the NHIRD analysis is insufficient to establish causality, leaving it uncertain whether the observed association represents a direct diabetogenic effect of oseltamivir or is instead attributable to the influenza infection itself. While previous studies have suggested potential mechanisms and documented instances of hyperglycemia following oseltamivir use, additional data and detailed pathway analyses are necessary to validate this hypothesis. Second, due to limitations in database availability, this study lacked baseline information on key indicators such as HbA1c levels, glucose levels, or medications for diabetes and related conditions. Additionally, undiagnosed conditions, such as underlying hypertension, may have led to underestimating its true prevalence and role in diabetes development. Third, this study did not assess whether oseltamivir dosage or repeated courses influence outcomes, though prolonged or repeated use might have cumulative effects on glucose metabolism. Moreover, the health status of patients and individual characteristics likely play a significant role in adverse effects. Patients prescribed oseltamivir may have more severe influenza or pre-existing comorbidities, potentially making them more prone to both influenza complications and Type 2 diabetes. This suggests that the observed association may reflect underlying health conditions rather than a causal link. Hence, we performed multivariate adjustments for comorbidities in the Cox proportional hazard regression models, utilized a large sample size, and applied 1:1 matching for age and gender to minimize bias. To further confirm the results, sensitivity analyses could be conducted by rerunning the analysis using methods like propensity score matching.

Another limitation is the inability to compare outcomes between oseltamivir-treated and untreated influenza patients due to the small number of untreated cases in the NHIRD, as oseltamivir is routinely prescribed for nearly all diagnosed influenza cases. Additionally, unmeasured factors such as lifestyle, concurrent medications, socioeconomic status, family history, and personal habits like smoking or alcohol consumption may act as confounders. However, the NHIRD lacks data on these factors. Instead, adjustments for related comorbidities such as obesity, hypertension, and dyslipidemia provide a rough estimate of their potential influence.

Despite its limitations, this study is one of the first to identify a correlation between antiviral agents and diabetes using a national health database. While the biological mechanisms remain unclear, the findings emphasize clinical relevance. Future studies should consider prospective cohort designs to track long-term metabolic outcomes in individuals prescribed oseltamivir, particularly for prophylaxis. Randomized controlled trials comparing oseltamivir with placebo in non-influenza contexts could help isolate the drug’s effects. The current retrospective design limits the establishment of causality; therefore, alternative approaches such as propensity score matching or instrumental variable analysis should be considered to strengthen causal inferences. Additionally, preclinical studies using animal models and in vitro systems should focus on oseltamivir’s impact on insulin signaling pathways, such as Neu-1 inhibition, and its downstream effects on glucose metabolism and beta-cell function. These approaches would help validate the hypothesized mechanisms and clarify the drug’s role in diabetes risk.

## 4. Materials and Methods

The Taiwan NHIRD, a single-payer system established in 1995, currently covers nearly 99% of the population. The program provides a comprehensive range of healthcare services, including outpatient care, inpatient services, dental care, medications, and surgical procedures. The NHIRD is one of the largest population-based databases globally, containing linked data such as demographic information, medical diagnoses, prescriptions, and claims from hospitals, clinics, and pharmacies. It has been extensively utilized for epidemiological research, with numerous studies published in peer-reviewed journals. The data for this study were obtained from the NHIRD, managed by Taiwan’s Ministry of Health and Welfare (grant no. H110290), and accessed via the Health and Welfare Data Science Center. To ensure privacy, all personal identifiers were anonymized, and as a result, informed consent was waived. The Institutional Review Board approved the study at Chung Shan Medical University Hospital (CS2-21180), and all data were analyzed at the Health and Welfare Data Science Center.

### 4.1. Oseltamivir Group

Patients who were prescribed oseltamivir between 1 January 2009 and 31 December 2018 were identified using relevant drug codes from the NHIRD (ATC: J05AH02, NHIRD codes: B024860100, B023253100). According to common practices and the health insurance payment guidelines in Taiwan, a diagnosis of influenza is typically followed by a standard therapeutic prescription of oseltamivir: 75 mg for adults, administered for 5 days. This regimen applies to nearly all patients. The date of the first prescription of oseltamivir was defined as the index date, and each patient’s age was determined at that time. Subsequent usage of oseltamivir was not monitored, limiting the ability to evaluate a potential dose-response relationship. To ensure study validity, individuals were excluded if they had multiple entries in the databases, unknown gender, and pre-existing DM diagnosis. Diagnosis of diabetes was confirmed if a single occurrence of a relevant ICD code (ICD-9: 250; ICD-10: E08-E13) was recorded in outpatient or inpatient settings before using oseltamivir.

### 4.2. Control Group

The control group consisted of patients who had not been prescribed oseltamivir during the same period (1 January 2009 to 31 December 2018). The same exclusion criteria were applied to both the oseltamivir and control groups to maintain comparability. Control patients were randomly selected and matched with oseltamivir patients based on exact age and gender to create a 1:1 match. Matching was performed using PROC PSMATCH Statement of SAS 9.4M8 software (SAS Institute Inc., Cary, NC, USA).

### 4.3. Outcome Determination

Diagnoses were identified using the International Classification of Diseases, 9th and 10th Revisions (ICD-9, ICD-10). The primary outcomes assessed included Type 1 diabetes (ICD-9: 250.x1, 250.x3; ICD-10: E10), Type 2 diabetes (ICD-9: 250.x0, 250.x2; ICD-10: E11), and diabetes of all types (ICD-9: 250; ICD-10: E08-E13). A diagnosis of diabetes was considered confirmed if a single occurrence of a relevant code was recorded in either outpatient or inpatient settings before the use of oseltamivir.

### 4.4. Comorbidities

The comorbidities assessed included dyslipidemia (ICD-9: 272), chronic liver disease, including cirrhosis (ICD-9: 571), hypertension (ICD-9: 401-405), coronary artery disease (CAD) (ICD-9: 410-414), cerebrovascular disease (ICD-9: 430-438), obesity (ICD-9: 278), chronic kidney disease (CKD) (ICD-9: 585), malignancy (ICD-9: 140-239), HIV infection (ICD-9: 041-044), and autoimmune diseases (ICD-9: 279.4). All comorbidities were identified by ICD-9 and ICD-10 codes and confirmed based on the criteria of at least three outpatient records or one inpatient record with the same diagnosis within 1 year before the index date.

### 4.5. Follow-Up

Patients were followed from the index date until they were diagnosed with any form of DM or until the end of the study period (31 December 2018).

### 4.6. Statistical Analysis

Continuous variables were expressed as mean and confidence interval (CI), while categorical variables were presented as counts and percentages. Pearson’s chi-squared tests were used to compare demographic differences between patients who used oseltamivir and those who did not. In subsequent analyses, Cox proportional hazard regression models were used to calculate the relative hazard ratio (HR) for developing DM, and multivariate analyses were conducted with adjustments for comorbidities. Each comorbidity’s relative hazard ratio (HR) was calculated and reported. This analysis indicates that, after adjusting for additional comorbidities and oseltamivir use, patients with a particular comorbidity exhibit an N-fold increased risk of developing diabetes compared to those without the specified comorbidity. A *p*-value < 0.05 was considered statistically significant. All statistical analyses were performed using SAS 9.4M8 software (SAS Institute Inc., Cary, NC, USA).

## 5. Conclusions

Utilizing longitudinal population-based data with up to 10 years of follow-up, this study revealed an association between oseltamivir use in influenza patients and an increased risk of developing diabetes, specifically Type 2, but not Type 1, in the general population of Taiwan. Our findings offer valuable insights into the potential metabolic implications of influenza treatment and the possible diabetogenic effects of oseltamivir. However, further research is required to establish causality and elucidate the underlying mechanisms.

## Figures and Tables

**Table 1:** Baseline characteristics of study participants.

Variable	Oseltamivir Use Group		Non-Oseltamivir Use Group		*p*-Value
	N	%	N	%	
Total	815,984	100%	815,984	100%	
**Gender**					1
Male	418,968	51.35	418,968	51.35	
Female	397,016	48.65	397,016	48.65	
**Comorbidities**					
Dyslipidemia	9697	1.19	44	0.01	<0001
Chronic liver disease	5499	0.67	10	0.00	<0001
Hypertension	20,249	2.48	57	0.01	<0001
CAD	4913	0.60	9	0.00	<0001
Cerebrovascular disease	2455	0.30	3	0.00	<0001
Obesity	536	0.07	4	0.00	<0001
CKD	1725	0.21	5	0.00	<0001
Malignancy	14,606	1.79	15	0.00	<0001
HIV infection	4273	0.52	4	0.00	<0001
Autoimmune disease	207	0.03	0	0.00	<0001
**Outcome**					
All DM	21,343	2.62	19,296	2.36	<0001
Type 1	895	0.11	832	0.10	0.1293
Type 2	20,627	2.53	18,647	2.29	<0001
**Follow time (years)**					
All DM (mean, SD)	6.8984	3.2223	6.9132	3.214	0.0035
Type 1	6.9979	3.1791	6.9983	3.1788	0.9247
Type 2	6.9023	3.2203	6.9166	3.2125	0.0045

N, Number; CAD, Coronary Artery Disease; CKD, Chronic Kidney Disease; HIV, Human immunodeficiency virus infection; DM, Diabetes Mellitus.

**Table 2:** Relative hazard ratio of the occurrence of diabetes and comorbidities in patients using oseltamivir compared to non-users.

	Non-Oseltamivir Use Group (HR)	Oseltamivir Use Group (HR)	95% CI (Low)	95% CI (Up)	*p*-Value
**All DM**	1 (reference)	1.027	1.004	1.05	0.0186
**Comorbidities**					
Dyslipidemia	1	1.295	1.18	1.421	<0001
Chronic liver disease	1	1.446	1.273	1.642	<0001
Hypertension	1	1.586	1.484	1.696	<0001
CAD	1	1.034	0.916	1.167	0.5888
Cerebrovascular disease	1	0.862	0.738	1.007	0.0608
Obesity	1	2.949	1.799	4.833	<0001
CKD	1	0.891	0.713	1.112	0.3065
Malignancy	1	0.968	0.881	1.064	0.5039
HIV infection	1	0.905	0.712	1.15	0.4129
Autoimmune disease	1	2.864	0.937	8.753	0.0649

DM, Diabetes Mellitus; CAD, Coronary Artery Disease; CKD, Chronic Kidney Disease; HIV, Human immunodeficiency virus infection.

**Table 3:** Relative hazard ratio of the occurrence of Type 1 diabetes and comorbidities in patients using oseltamivir compared to non-users.

	Non-Oseltamivir Use Group (HR)	Oseltamivir Use Group (HR)	95% CI (Low)	95% CI (Up)	*p*-Value
**Type 1 DM**	1 (reference)	1.021	0.925	1.127	0.6795
**Comorbidities**					
Dyslipidemia	1	1.497	0.733	3.059	0.2684
Chronic liver disease	1	1.817	0.73	4.523	0.1997
Hypertension	1	1.78	1.047	3.024	0.0331
CAD	1	0.687	0.232	2.03	0.4971
Cerebrovascular disease	1	0.629	0.206	1.914	0.4139
Obesity	1	>10	<0.001	>100	0.9713
CKD	1	1.394	0.335	5.8	0.6476
Malignancy	1	1.19	0.634	2.232	0.5888
HIV infection	1	7.578	0.948	60.558	0.0562
Autoimmune disease	1	N/A	N/A	N/A	N/A

DM, Diabetes Mellitus; CAD, Coronary Artery Disease; CKD, Chronic Kidney Disease; HIV, Human immunodeficiency virus infection; N/A, not applicable.

**Table 4:** Relative hazard ratio of the occurrence of Type 2 diabetes and comorbidities in patients using oseltamivir compared to non-users.

	Non-Oseltamivir Use Group (HR)	Oseltamivir Use Group (HR)	95% CI (Low)	95% CI (Up)	*p*-Value
**Type 2 DM**	1 (reference)	1.024	1.001	1.048	0.037
**Comorbidities**					
Dyslipidemia	1	1.306	1.188	1.434	<0001
Chronic liver disease	1	1.463	1.286	1.664	<0001
Hypertension	1	1.607	1.502	1.72	<0001
CAD	1	1.032	0.913	1.166	0.6167
Cerebrovascular disease	1	0.84	0.718	0.983	0.0296
Obesity	1	2.8	1.704	4.6	<0001
CKD	1	0.895	0.715	1.121	0.334
Malignancy	1	0.959	0.872	1.055	0.3922
HIV infection	1	0.891	0.698	1.137	0.3535
Autoimmune disease	1	2.87	0.939	8.775	0.0644

DM, Diabetes Mellitus; CAD, Coronary Artery Disease; CKD, Chronic Kidney Disease; HIV, Human immunodeficiency virus infection.

## Data Availability

The data presented in this study are available on request from the corresponding author. The data are not publicly available due to privacy and ethical restrictions.
